# The Clinical Value of NT-proBNP, sST2, and Galectin-3 in the Management of Heart Failure: From Diagnosis to Dynamic Monitoring: A Narrative Review

**DOI:** 10.3390/ijms27167480

**Published:** 2026-08-21

**Authors:** Diana Andreea Fărcaș, Monica Tarcea, Maria Cristina Tătar, Anda Cerghizan, Călin Crăciun, Hajnal Finta, Florina Gliga, Claudia Bănescu

**Affiliations:** 1 Doctoral School of Medicine and Pharmacy, George Emil Palade University of Medicine, Pharmacy, Science, and Technology of Târgu Mureș, 540139 Târgu Mureș, Romania; 2 Emergency Institute for Cardiovascular Diseases and Transplantation of Târgu Mureș, 540136 Târgu Mureș, Romania; 3 Department of Community Nutrition and Food Safety, George Emil Palade University of Medicine, Pharmacy, Science, and Technology of Târgu Mureș, 540139 Târgu Mureș, Romania; monica.tarcea@umfst.ro; 4 Department of Clinical and Surgical Disciplines, Faculty of Medicine, George Emil Palade University of Medicine, Pharmacy, Science, and Technology of Târgu Mureș, 540142 Târgu Mureș, Romania; maria.tatar@umfst.ro; 5 Department of Surgery III, Faculty of Medicine, George Emil Palade University of Medicine, Pharmacy, Science, and Technology of Târgu Mureș, 540139 Târgu Mureș, Romania; calin.craciun@umfst.ro; 6 Department of Public Health and Management, Faculty of Medicine, George Emil Palade University of Medicine, Pharmacy, Science, and Technology of Târgu Mureș, 540139 Târgu Mureș, Romania; hajnal.finta@umfst.ro; 7 Department of Physiopathology, Faculty of Medicine, George Emil Palade University of Medicine, Pharmacy, Science, and Technology of Târgu Mureș, 540139 Târgu Mureș, Romania; florina.gliga@umfst.ro; 8 Department of Medical Genetics, George Emil Palade University of Medicine, Pharmacy, Science, and Technology of Târgu Mureș, 540139 Târgu Mureș, Romania; claudia.banescu@umfst.ro

**Keywords:** sST2, Gal-3, heart failure, NT-proBNP

## Abstract

Heart failure management remains a critical clinical challenge. While modern neurohormonal therapies have significantly improved survival, standard clinical assessments often fail to capture the full complexity of underlying structural disease progression. To comprehensively synthesize the established literature regarding the biological and prognostic roles of NT-proBNP, soluble ST2 (sST2), and Galectin-3 (Gal-3) and, subsequently, to propose a hypothesis-generating multimarker framework for HF risk stratification. A narrative review of the literature (2006–2026) was conducted across PubMed, Scopus, and Google Scholar. The synthesis focused on the clinical utility, phenotypic specificities, and methodological limitations of evaluating NT-proBNP, sST2, and Gal-3 concentrations. Evidence from the literature confirms that while NT-proBNP remains the gold standard for acute hemodynamic assessment. sST2 and Gal-3 provide complementary insights into active biomechanical strain and interstitial fibrotic remodeling; however, prospective randomized clinical trials demonstrating that sST2- or Gal-3-guided management improves clinical outcomes remain lacking. Based on these established findings, we propose an investigational four-step multimarker framework spanning from acute diagnosis to dynamic outpatient monitoring. This framework hypothesizes that integrating these pathways can better identify high-risk phenotypes that remain undetected by natriuretic peptides alone. The synergistic application of NT-proBNP, sST2, and Gal-3 offers a deeper pathophysiological evaluation of HF. However, our proposed biomarker-guided framework represents a set of testable research hypotheses. It requires prospective randomized validation before integration into routine clinical protocols for therapeutic titration.

## 1. Introduction

Heart failure (HF), despite the remarkable advances in therapy over the past few decades, remains one of the most common causes of hospitalization. It is not just a single disease, but a complex systemic process with many causes [1]. Chronic inflammation and fibrous remodeling are the two main pathophysiological mechanisms. Specifically, this fibrotic remodeling is driven by specialized pathways involving molecular mediators of the extracellular matrix, such as Gal-3 (which mediates macrophage activation and fibroblast proliferation) and procollagen turnover peptides (e.g., serum N-terminal pro-peptide of procollagen type III [PIIINP]). These specific fibrotic markers are essential, as they reflect the active transition from adaptive hypertrophy to irreversible myocardial stiffening and interstitial expansion [2,3,4].

Heart failure is more than just a diagnosis. It is a complex syndrome characterized by symptoms such as dyspnea, fatigue, and systemic congestion and can lead to multiple organ failure [5,6].

Knowing the causes is important to guide treatment based on the underlying cause. Most often, the main causes are coronary artery disease in developing countries and valvular pathology, a progressive age-related disease. In World Health Organization (WHO) countries, the prevalence of rheumatic heart disease remains high [7]. These patients are also more likely to suffer from heart failure with preserved fraction of ejection (HFpEF), which is often underdiagnosed. In addition, congenital malformations, hypertension, obesity, and arrhythmias are important factors to consider, and these patients are more likely to have HFpEF, which is mostly underdiagnosed [8,9,10].

According to HF Stats 2024, heart failure affects 6.7 million Americans over 20 years old and is expected to reach 10.4 million in 2050 [11]. In Europe, between 15 and 20 million people are registered, and the percentage of people with HFpEF is increasing [12]. According to the Romanian Society of Cardiology and National Health Strategy 2021–2027, the prevalence in Romania is 4.7% and is expected to reach 5% in 2030 [13,14]. Considering the increasing prevalence and importance of heart failure in the world population, it is important to develop rapid diagnostic methods and implement risk stratification. Investigations, treatment, and treatments are also needed [8,12].

Serum biomarkers are the most convenient and quantifiable elements in the diagnosis and therapy of heart failure. Currently, BNP, NT-proBNP, and troponin are the most widely used biomarkers. They provide initial information on the patient’s health. The greatest disadvantage is the association with other conditions such as age, chronic renal disease, atrial flutter, and overweight. In the outpatient setting, the European Society of Cardiology guidelines outline non-acute diagnostic exclusion cut-offs—specifically 35 pg/mL for BNP and 125–300 pg/mL for NT-proBNP—which must be interpreted cautiously alongside clinical probability and echocardiography, as levels are heavily confounded by age, renal function, and body mass index [5].

The purpose of this review is to synthesize the available/current evidence on the biological roles of NT-proBNP, soluble sST2, and Gal-3 in the management of heart failure to propose a four-step biomarker-guided algorithm designed to optimize risk stratification and therapeutic titration and monitoring.

## 2. Materials and Methods

To develop our narrative review, a comprehensive literature search was conducted in major electronic databases, including PubMed, Scopus, and Google Scholar between 2006 and 2026. To ensure full retrieval of the most recent evidence, the final search update was executed on 1 August 2026. The search strategy focused on identifying high-impact clinical trials, meta-analyses and consensus articles in English that evaluate the clinical utility of sST2, Gal-3 and NT-proBNP, as well as the multimarker approach in the management of heart failure.

In the literature searching strategy, we used specific database Boolean operators (AND, OR) and MeSH terms. Filters applied across databases were: publication date between 1 January 2006 and 15 June 2026, English language, and peer-reviewed human clinical studies.

The database-specific search keywords were as follows:

PubMed: ((“heart failure”[MeSH Terms] OR “heart failure”[All Fields]) AND (“NT-proBNP”[All Fields] OR “N-terminal pro-B-type natriuretic peptide”[All Fields]) AND (“sST2”[All Fields] OR “soluble ST2”[All Fields] OR “IL1RL1”[All Fields]) AND (“Galectin-3”[All Fields] OR “GAL-3”[All Fields]) AND (“prognosis”[All Fields] OR “diagnosis”[All Fields] OR “monitoring”[All Fields])).

Scopus: TITLE-ABS-KEY ((“heart failure”) AND (“NT-proBNP” OR “N-terminal pro-B-type natriuretic peptide”) AND (“sST2” OR “soluble ST2”) AND (“Galectin-3” OR “Gal-3”) AND (“prognosis” OR “diagnosis” OR “monitoring”)) AND PUBYEAR > 2005 AND PUBYEAR < 2027 AND (LIMIT-TO (LANGUAGE, “English”)).

Google Scholar: A targeted search using the query “NT-proBNP” AND “sST2” AND “Galectin-3” AND “heart failure” yielded 791 total records. The results were sorted by relevance and the first 200 records (first 20 pages) were systematically screened for full-text eligibility.

A comprehensive literature search in PubMed, Scopus, and Google Scholar yielded a total of 288 records selected for screening. The specific database contributions were as follows: PubMed (*n*= 45), Scopus (*n* = 43), and Google Scholar (*n* = 200; specifically, the first 20 pages of the most relevant records were screened out of 791 total hits to ensure high-quality retrieval).

After removing 24 duplicate records, 264 unique records were screened by title and abstract, resulting in the exclusion of 162 irrelevant studies. The remaining 102 full-text articles were thoroughly assessed for eligibility. Among these, 70 were excluded for specific reasons: insufficient quantitative data (*n* = 57), failure to meet the inclusion criteria (*n* = 11), and inaccessible full text in English (*n* = 2). Ultimately, 32 original studies satisfied all inclusion criteria and were incorporated into the review synthesis.

Inclusion criteria were as follows: (1) adult patients diagnosed with heart failure, regardless of baseline NYHA functional class; (2) peer-reviewed original clinical studies and meta-analyses published in English (2006–2026) evaluating multiple markers or a single strategy that includes sST2, Gal-3 and NT-proBNP.

Exclusion criteria: Animal/in vitro studies, pediatric populations (<18 years), non-peer-reviewed sources (preprints, conference abstracts, editorials) and studies with incomplete biomarker or clinical outcome data.

No assessment or risk-of-bias was performed for the included studies, which represents a primary methodological limitation of this review.

While all 32 studies informed our comprehensive clinical synthesis, Supplementary Table S1 deliberately highlights only the most representative, pivotal landmark studies that shaped our proposed framework, keeping in line with the narrative scope of this review.

## 3. NT-proBNP

### 3.1. Pathophysiology and Mechanisms

NT-proBNP is part of a neurohormone class derived from proBNP, a member of the natriuretic peptide family, released by the heart under conditions of stress, high filling pressure, and volume overload. The family of natriuretic peptides includes ANP (atrial natriuretic peptide), which is secreted by atrial myocytes, BNP, which is secreted by ventricular myocytes and the C-type natriuretic peptide. BNP, the active form found in serum, was first identified in 1988 in the brain of pigs, but subsequent studies showed that it is secreted by ventricular myocytes. Given a stress condition, proBNP binds to BNP, the biologically active part with a very short half-life, and NT-proBNP, the inactive part with a prolonged half-life [14,15]. The heart synthesis of pre-proBNP in myocytes is proportional to the severity of the dysfunction and responds to mechanical and neurohormonal mechanisms after overload and high pressure. After its release, BNP leads to vasodilation, increased diuresis, and inhibition of the renin–angiotensin–aldosterone system (RAAS), whereas NT-proBNP remains highly stable and accumulates in the circulation. NT-proBNP undergoes renal elimination, which partially explains why its concentrations depend on renal function [16,17,18,19,20].

### 3.2. Diagnostic Evidence

NT-proBNP is the most widely used biomarker in emergency diagnosis and chronic patient management. Rasmus Rørth et al. highlighted the “6.25” paradigm—the NT-proBNP/BNP ratio in the general population—which is higher than mentioned in the current guidelines. This ratio varies with associated diseases such as atrial fibrillation and chronic kidney disease, as well as with age, particularly in the elderly [20]. A multicenter study highlights that NT-proBNP is the gold standard for the initial evaluation of dyspneic patients; a cut point of <300 pg/mL yields 96% sensitivity and a negative predictive value of 95% [21,22,23].

Furthermore, the ICON RELOADED study (2020) stresses that the threshold for NT-proBNP to rule out heart failure is 300 pg/mL (measured via standard chemiluminescence immunoassays). However, diagnostic cut-offs for ruling in acute heart failure must be adjusted (Table 1) [24].

Importantly, NT-proBNP varies by demographic factors; it is higher in women, increases with age, and its association with heart failure risk is greater in white individuals compared to black individuals [25,26,27]. The main diagnostic limitation of NT-proBNP is that it reflects mechanical stress exclusively, ignoring parallel pathological processes like myocardial fibrosis or systemic inflammation, prompting a shift toward multimarker diagnostic models [28].

### 3.3. Prognostic Evidence

NT-proBNP is a powerful prognostic factor in both HFrEF and HFpEF. In PARADIGM-HF, NT-proBNP successfully identified patients with higher risk and predicted milestone events, including electrical instability. A cohort study of 500 ICD patients found baseline NT-proBNP was strongly associated with ventricular arrhythmia risk (HR: 1.39, *p* < 0.001), heart failure hospitalizations (HR: 3.11), and all-cause mortality (HR: 2.49), independent of comorbidities [29,30,31]. Additionally, NT-proBNP is inversely associated with myocardial recovery; for each 1-unit increase in its square root, the probability of recovery decreases.

Patients with de novo heart failure and values >10,000 pg/mL have a <20% chance of recovering ejection fraction after discharge [32]. Chevalier et al. also reported a high correlation with diastolic dysfunction and structural modifications in hypertrophic cardiomyopathy [33]. Emerging genetic data (Long Life Family Study) and genetic risk scores developed by Yang et al. show that NT-proBNP levels and cardiovascular risk are heavily influenced by genetics and socioeconomic status, opening the door for precision medicine [34,35,36].

### 3.4. Predictive Evidence

Predictive evidence highlights the ability of the biomarker to guide specific therapeutic interventions. The recent STRONG HF study of 1000 patients with acute heart failure outlined that NT-proBNP serves as a highly effective guide for the titration of guideline-recommended therapies in high-intensity care. Orienting therapy according to these levels resulted in significantly lower readmission rates and cardiovascular deaths [37,38].

### 3.5. Serial-Monitoring Evidence

In the context of long-term serial monitoring, pooled data from 12 clinical trials in patients with HFrEF demonstrate that the use of natriuretic peptides for the continuous adjustment of treatment directly affects survival. The NT-proBNP-monitored group experienced a mortality rate of 14.7% compared with 18.1% in the control group. Although prognostic accuracy may slightly decrease in patients >75 years of age, serial assessment of NT-proBNP remains superior to any other single biomarker method for tracking mortality risk over time [30].

## 4. The New Challenger—sST2 (Soluble Suppression of Tumorigenicity)

### 4.1. Pathophysiology and Mechanisms

**Figure 1:**
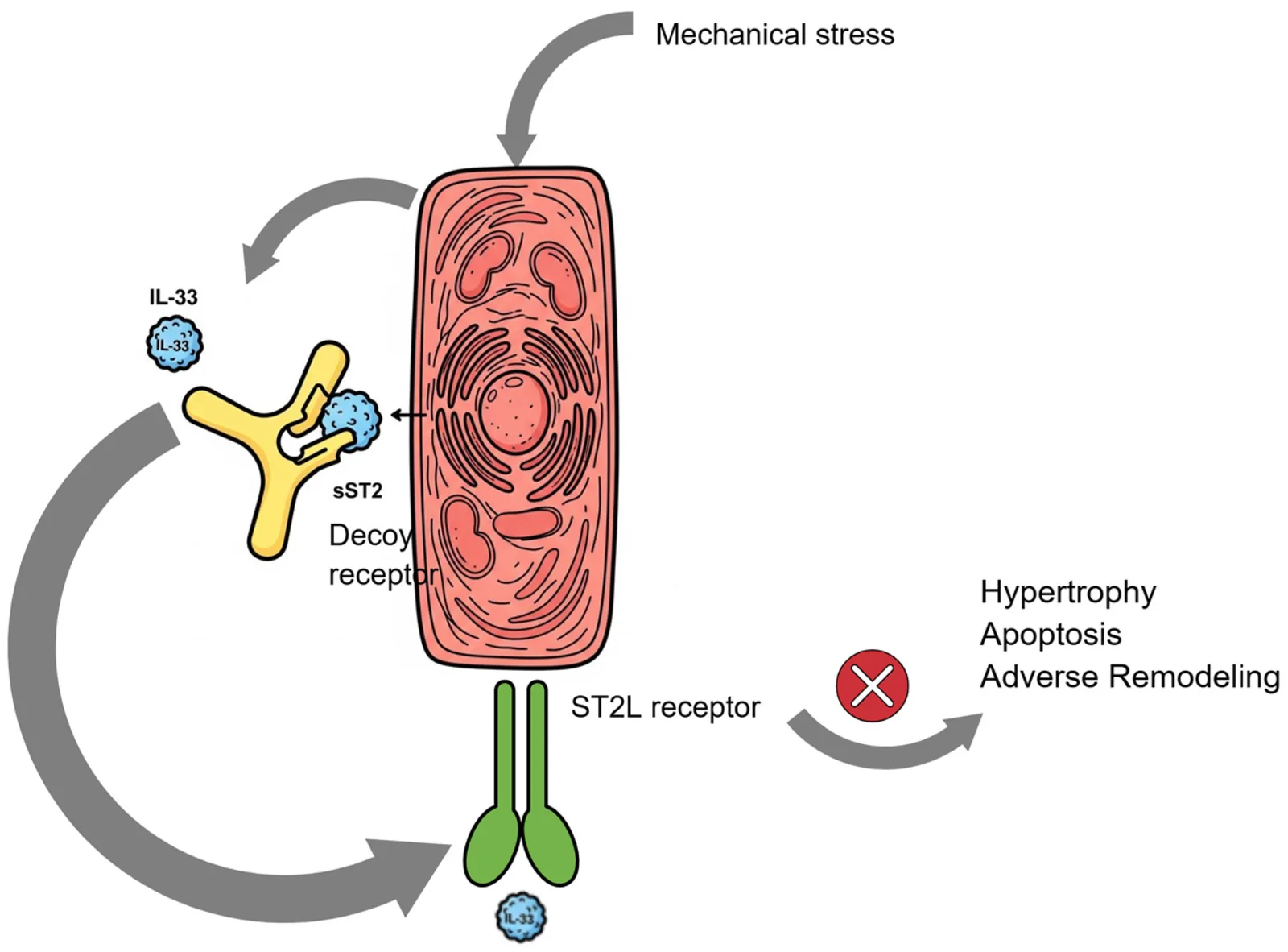
Pathophysiological mechanism of sST2.

Suppression of tumorigenicity 2 (ST2), coded by IL1RL1, is part of the interleukin 1 receptor family. It exists as a transmembrane isoform (ST2L) and a soluble form (sST2), both expressed in cardiac myocytes and fibroblasts. Interleukin-33 (IL-33) is a mechanosensitive alarmin produced in response to cellular stress. The combination ST2L/IL-33 exerts a cardioprotective effect, reducing myocardial fibrosis, cardiomyocyte hypertrophy, and apoptosis [39]. However, sST2 acts as a “decoy” receptor, competing with ST2L for IL-33. When sST2 binds to IL-33, it blocks the functional ST2L/IL-33 pathway, eliminating these cardioprotective effects (Figure 1). Consequently, the ST2 system modulates both beneficial and maladaptive structural outcomes through IL-33 regulation [40,41,42].

### 4.2. Diagnostic Evidence

The expression of sST2 is induced by inflammatory and fibrotic responses and mechanical lengthening [43]. Unlike NT-proBNP, which reflects acute hemodynamic pressure, sST2 provides a diagnostic window into active ventricular remodeling and fibrosis. Because it reflects the disruption of the cardioprotective IL-33/ST2L pathway under pro-fibrotic stress, it holds significant potential as a prognostic marker for risk stratification [44]. However, its diagnostic performance is limited by a lack of organ specificity. sST2 is an active component of the inflammatory cascade in pulmonary or autoimmune pathologies, which can generate false-positive results in complex comorbidities [45,46].

### 4.3. Prognostic Evidence

ST2 is no longer an experimental marker but an important prognostic tool, included in the 2017 AHA Guidelines for additive risk stratification [47]. Post hoc analyses of PRAISE first identified high sST2 levels as an independent predictor of all-cause mortality or transplantation [48]. Meta-analyses confirm a Hazard ratio of 1.50 for major cardiovascular events in all subgroups, regardless of ethnicity or LVEF [49]. Compared with NT-proBNP, sST2 is less dependent on glomerular filtration rate, retaining good prognostic value in cardiorenal syndrome, though advanced renal dysfunction remains an important clinical confounder [50,51].

Furthermore, concentration thresholds, such as values exceeding 35 pg/mL (measured via the Presage ST2 assay in both acute discharge and chronic stable cohorts) or higher-risk ranges approaching 70 pg/mL, can help flag patients at increased risk for adverse post-infarction remodeling or a pro-arrhythmic atrial substrate, serving as valuable prognostic indicators within a comprehensive risk-stratification approach [49].

The BIOS consortium established gender-specific prognostic thresholds, acknowledging that natural sST2 concentrations are lower in women (Table 2) [52,53].

### 4.4. Predictive Evidence

Data from the PARADIGM-HF trial highlight sST2 as a valuable tool for assessing the therapeutic response and efficacy of sacubitril/valsartan. Furthermore, unlike natriuretic peptides, sST2 demonstrates better stability across varying body mass indices, providing independent prognostic assessment unaffected by BMI variations or baseline fluctuations in NT-proBNP and hs-TnT [31].

### 4.5. Serial-Monitoring Evidence

A major paradigm shift is the move from single measurements to serial monitoring. Dynamic changes in sST2 are more predictive of 90-day mortality than baseline values [47]. The RELAX-AHF trial demonstrated that serial sST2 assessment at 14 and 60 days post-discharge provides critical prognostic insights and persistently high levels may signal residual risk and a suboptimal therapeutic response, helping guide timely clinical reevaluation before overt symptoms reappear [54,55].

## 5. Gal-3—The Fibrosis Footprint

### 5.1. Pathophysiology and Mechanisms

**Figure 2:**
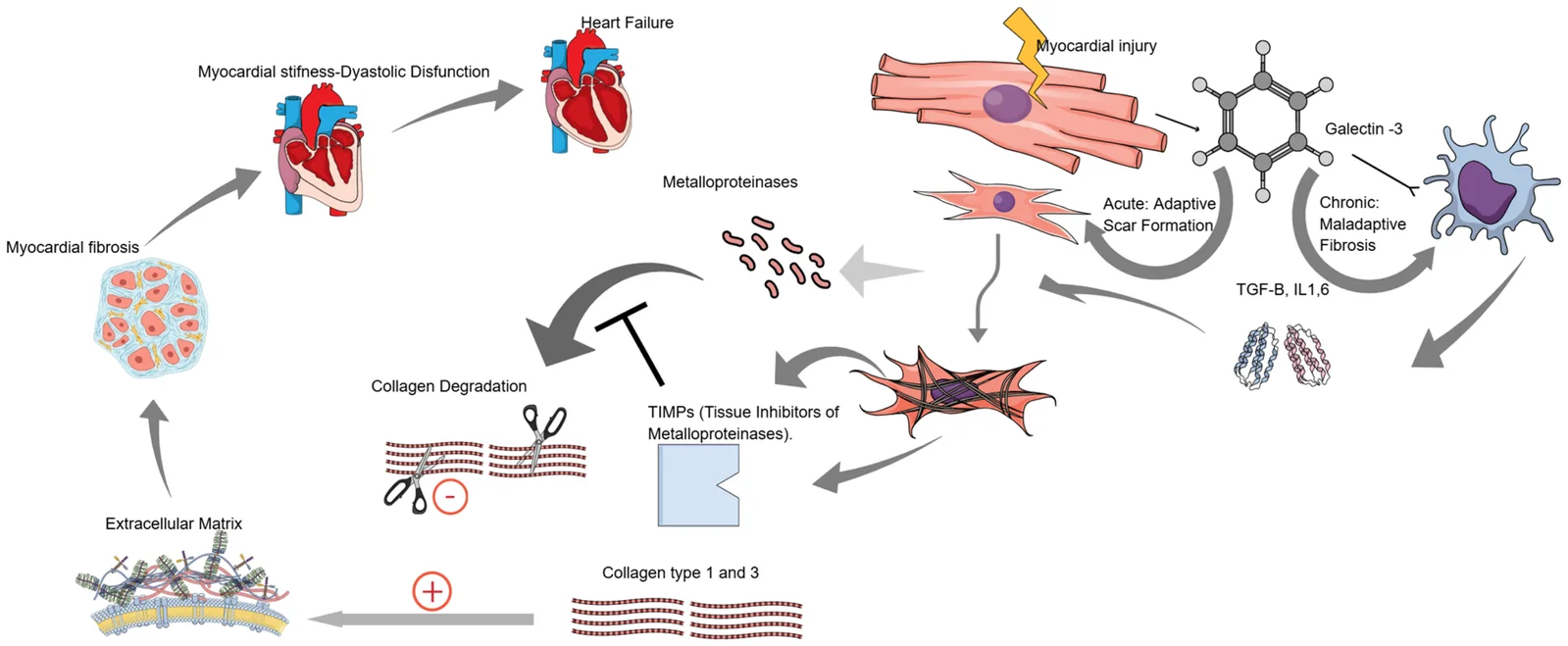
The Gal-3 pathological signaling cascade in cardiac fibrosis [2,3].

Gal-3, a beta-galactoside-binding lectin, represents the missing piece in understanding cardiac remodeling. Its serum levels do not only reflect pressure responses but also the active initiation of the fibrotic cascade. It mediates macrophage activation and fibroblast proliferation, directly driving collagen deposition in the extracellular matrix (Figure 2) [56]. Its role is biphasic: in the acute phase of myocardial infarction, Gal-3 exerts a protective effect essential for wound healing. However, persistent Gal-3 secretion in the late phase induces chronic inflammation and interstitial fibrosis, accelerating adverse ventricular remodeling [57].

### 5.2. Diagnostic Evidence

Gal-3 functions as a structural phenotyping tool capable of detecting preclinical diastolic dysfunction. It correlates directly with the severity of diastolic dysfunction (E/e′ index) and is superior to NT-proBNP for the early diagnosis of HFpEF in asymptomatic patients (C-index 0.64 vs. 0.62 for BNP) [57]. It also correlates strongly with right ventricular pressure and actively contributes to pulmonary hypertension through the NOX4 pathway [58]. However, as a diagnostic marker for acute heart failure decompensations, Gal-3 has a lower sensitivity and specificity than NT-proBNP. It does not correlate linearly with the severity of current symptoms (NYHA class) because macrophage-mediated fibrogenesis does not fluctuate with immediate volume changes [59].

### 5.3. Prognostic Evidence

Gal-3 is an established predictor of disease progression and mortality. In the acute phase, it carries a Hazard ratio of 2.17 for mortality (compared to 1.13 in chronic forms) and a Relative Risk of 1.29 for cardiovascular mortality [60,61]. Aggregate analyses (COACH, PRIDE, UMD H-23258) identified a prognostic threshold of 17.8 pg/mL (evaluated at discharge in acute heart failure patients) to discriminate high-risk patients for early rehospitalization. Interestingly, in the SHOCK-COOL trial for cardiogenic shock, a threshold below 3.65 pg/mL predicted 30-day mortality, suggesting an immune system failure to initiate post-ischemia repair [62,63,64].

Clinical Limitations on Prognostic Thresholds: The prognostic value of Gal-3 can be confounded by non-cardiac conditions. Gal-3 overestimates risk in patients with low eGFR, cirrhosis, or pulmonary fibrosis [2,65]. In addition, another category limited by Gal-3 testing is obese patients. Because Gal-3 is actively secreted by macrophages infiltrating white adipose tissue, its circulating levels are directly correlated with body mass index (BMI) and waist circumference. Therefore, in this group of patients, the risk threshold should be interpreted with caution, as Gal-3 reflects systemic metabolic inflammation rather than isolated cardiac remodeling [66].

### 5.4. Predictive Evidence

The AHA/ACC/HFSA guidelines assign Gal-3 a Class IIa recommendation for risk stratification. When integrated into a multimarker approach, Gal-3 serves as a valuable indicator of myocardial fibrotic remodeling. While sST2 reflects acute myocardial stress and helps assess therapeutic efficacy, Gal-3 identifies the presence of established chronic fibrosis, thereby refining long-term risk assessment and mitigating diagnostic interference from transient biomarker fluctuations [67,68,69,70,71].

### 5.5. Serial-Monitoring Evidence

Gal-3 serves as a valuable tool for longitudinal tissue characterization. Emerging evidence demonstrates a significant correlation between serially monitored Gal-3 levels and myocardial structural remodeling, encompassing both diffuse interstitial fibrosis assessed by T1 mapping and focal scarring detected by late gadolinium enhancement (CMR). This underscores its utility as a biomarker for monitoring continuous myocardial changes over time [71].

## 6. The Multimarker Approach

Modern management of heart failure is increasingly based on multimarker strategies. Although NT-proBNP remains the standard for diagnosing and monitoring hemodynamic decompensation, integrating biomarkers that reflect underlying structural processes provides a more comprehensive assessment of disease progression. This approach, initially conceptualized by Braunwald in 2008, classifies biomarkers into categories such as inflammation, wall stress, myocyte injury, and extracellular matrix remodeling (Table 3) [72].

We specifically focused on the triad of NT-proBNP, Gal-3, and sST2 to provide a reliable approach to heart failure assessment. As noted in the comprehensive review by Alzaabi et al., many novel biomarkers such as cardiac troponins, CRP, IL-6, Cystatin C, Creatinine, and MicroRNAs still face significant limitations in clinical practice, such as a lack of standardization and insufficient evidence. Therefore, NT-proBNP is affirmed as the diagnostic standard for hemodynamic stress and combined with Gal-3 and sST2, which the referenced article highlights as indicators of myocardial fibrosis and inflammation. By limiting our scope to these three established markers, our review avoids purely experimental tools and instead delivers a targeted analysis of biomarkers that offer recognized value for risk stratification and prognostic evaluation, while acknowledging their inherent clinical limitations [73].

This strategy allows for the evaluation of three distinct complementary pathophysiological domains:

- Hemodynamic pathway (NT-proBNP): This indicates ventricular myocyte stretch in response to volume and pressure overload.
- Remodeling pathway (sST2): This reflects the neutralization of cardioprotective interleukin-33, exposing the myocardium to mechanical stress.
- Fibrosis pathway (Gal-3): This signals fibroblast activation and collagen deposition in the extracellular matrix.

**Table 3:** Biomarker profile in heart failure.

	NT-proBNP	sST2	Gal-3
Physiopathology	Reflects parietal tension and myocyte stretching [29,74,75].	Reflects the failure of myocardial adaptation by sequestering IL-33 [39,42,75]	Reflects fibroblast activation with extracellular matrix formation [2,3,74]
Diagnostic Role	Gold standard for acute rule-in and rule-out of heart failure [29,75].	Not recommended for the primary diagnosis of heart failure.	Not recommended for primary diagnosis of heart failure.
Prognostic Role	Strong predictor of mortality and rehospitalization in all heart failure phenotypes.	A robust independent predictor of death 30 days after discharge [29,76].	An indicator of chronic progression, incident heart failure, and irreversible fibrosis [2,3,74]
Monitoring Evidence	Provides rapid calibration of the current volume status; fluctuates rapidly depending on diuretic treatment [29,75].	Serial changes help reclassify risk when NT-proBNP is unclear; indicates reverse remodeling [29,39,42,76].	Reflects long-term structural changes (less dynamic); monitors the degree of myocardial fibrosis [2,3,74].
Confounders	Strongly influenced by age, obesity (lower levels), renal dysfunction and atrial fibrillation (higher levels) [77].	Less influenced by age, sex, or renal function compared to NT-proBNP; however, levels can be elevated in systemic inflammatory states or sepsis [39,42,76].	Strongly influenced by renal function (reduced clearance) and other fibrotic diseases (e.g., liver or pulmonary fibrosis).
Assay Limitations	The results must be adjusted for age; renal impairment significantly alters the clearance and interpretation.	Intra-individual biological variation exists; careful standardization is required between different immunoassay kits.	Highly dependent on estimated glomerular filtration rate; renal function must be evaluated constantly.
Guideline Status	Class I, Level of Evidence A (ESC/AHA) for both diagnosis and prognosis.	Observationally supported for risk stratification. There are no specific Class/LOE in the current guidelines for the up-titration of routine therapy.	Observationally supported for risk stratification. No specific Class/LOE in current guidelines for routine therapy up-titration.

### 6.1. Phenotype and Setting Differentiation: Acute vs. Chronic and HFrEF vs. HFpEF

The utility of these biomarkers varies significantly depending on the clinical setting (acute or chronic) and the heart failure phenotype (HFrEF vs. HFpEF). In the context of HFpEF, Cui et al. demonstrated that Gal-3 shows superior discriminatory capacity compared to sST2, producing an area under the receiver operating characteristic curve (AUC) of 0.819 at a cut-off of 9.55 pg/mL. Furthermore, Gal-3 remains a robust predictor of 1-year adverse events specifically in HFpEF cohorts, maintaining its predictive power after adjusting for NT-proBNP. This is supported by its correlation with the E/e′ ratio (r = 0.153), a key echocardiographic index of diastolic dysfunction and myocardial stiffness [78].

In contrast, in HFrEF, sST2 appears to offer greater prognostic utility. It strongly correlates with decreased ejection fraction (r = −0.57) and predicts adverse outcomes with an HR of 2.36 [78,79].

Further evidence demonstrates broad utility across phenotypes. For example, Rehman et al. reported that the prognostic value of sST2 was independent of left ventricular ejection fraction, supporting its applicability across the entire spectrum of heart failure [78]. Regarding the clinical setting, Mueller et al. established that sST2 and Gal-3 are not appropriate for the acute diagnosis of heart failure. Instead, their value lies in chronic prognostication and post-discharge monitoring, offering complementary risk assessment to natriuretic peptides for medium- to long-term mortality [80]. A meta-analysis of 20 studies confirmed a linear relationship between Gal-3 and adverse outcomes, reporting an HR of 1.65 for all-cause mortality and 1.52 for cardiac rehospitalization, with a one-log unit increase in Gal-3 significantly elevating mortality risk [81].

### 6.2. The Interplay Between Obesity, Ventricular Dynamics, and Natriuretic Peptide Suppression in HFpEF

Heart failure phenotypes differ significantly based on left ventricular ejection fraction, with distinct pathophysiological trajectories and diagnostic challenges. In heart failure with preserved ejection fraction specifically, the left ventricle typically undergoes concentric hypertrophy rather than cavity dilatation. Because significant left ventricle dilatation is absent, the mechanical wall stretch that normally triggers natriuretic peptide (NP) release is less pronounced, making biomarkers like NT-proBNP slightly less predictive than in reduced ejection fraction phenotypes [82].

In this specific context, obesity plays a disproportionately important role. The relationship between excess adiposity and cardiac dysfunction extends beyond simple mechanical overload, involving complex interactions that directly alter left ventricle dynamics. Excess adipose tissue, particularly visceral and epicardial adiposity, acts as an active endocrine organ. Adipocyte hypertrophy promotes chronic low-grade inflammation, releasing pro-inflammatory adipokines that induce endothelial dysfunction and cardiac fibroblast activation. This results in myocardial fibrosis, which further increases ventricular stiffness and impairs diastolic relaxation without necessarily dilating the ventricle. Arterial hypertension acts as an additional pivotal mediator on this axis, contributing substantially to the increase in left ventricular mass and non-ischemic cardiomyopathy progression [83].

This altered left ventricle dynamic is further complicated by the obesity–natriuretic peptide paradox. While HFpEF alone limits natriuretic peptide elevation, adiposity actively suppresses circulating levels even further, despite increased hemodynamic risk and severe ventricular wall stress. This paradox is driven by two main mechanisms. First, expanded adipose tissue expresses increased densities of clearance receptors, which capture and degrade circulating peptides. Second, the hyperinsulinemia and insulin resistance characteristic of obesity alter cellular energy pathways and suppress the intrinsic production of natriuretic peptides in cardiomyocytes. Consequently, the metabolic environment of the obese patient masks the true severity of the disease through falsely low natriuretic peptide values, contrasting sharply with states of cachexia where biomarker levels are markedly elevated [83,84].

These combined anatomical and metabolic particularities complicate the application of standardized diagnostic algorithms, such as HFA-PEFF and H2FPEF. To avoid the underdiagnosis of HFpEF in obese patients, the decision cut-offs for NT-proBNP and BNP must be significantly reduced. Furthermore, indexing the left atrial volume and left ventricular mass to body surface area can underestimate the degree of remodeling due to aberrantly high values in obesity, making indexing to height highly recommended. When adipose tissue degrades the echocardiographic window and limits the accuracy of classic measurements, evaluating myocardial deformation through global longitudinal strain or utilizing cardiac magnetic resonance becomes essential. Together, these elements underscore the need for a personalized approach to risk stratification and heart failure diagnosis in the obese patient, carefully integrating specific left ventricle ejection fraction phenotypes, altered dynamics, and a highly nuanced interpretation of inherently lower molecular biomarkers [82,83,84].

### 6.3. Incremental Value Beyond Standard Clinical Models

To be clinically viable, new biomarkers must demonstrate an incremental prognostic value beyond established parameters, including NT-proBNP, high-sensitivity troponin, estimated glomerular filtration rate, echocardiography, and validated clinical scores (e.g., MAGGIC or the Seattle Heart Failure Model). sST2 fulfills several of these criteria. It is less influenced by renal function (eGFR) than NT-proBNP and correlates significantly with high-sensitivity C-reactive protein (hsCRP, r = 0.163), capturing inflammatory risk not assessed by natriuretic peptides [78].

Machine learning models highlight this incremental value: while individual markers such as sST2 (AUC 0.711) or Gal-3 (AUC 0.777) show modest independent discriminatory capacity, a combined multimarker model (BNP, sST2, Gal-3, and CK-MB) achieved 91.5% sensitivity and 96.7% specificity for identifying high-risk early-stage heart failure [85].

Furthermore, serial testing provides significant reclassification value. In outpatient cohorts with chronic heart failure, an sST2 ratio (value at two weeks divided by baseline) of >0.75 indicates a failure to stabilize remodeling processes and strongly predicts adverse events. Conversely, a >25% reduction in sST2 over two weeks is a favorable prognostic indicator. The highest risk cohort (72% event rate) consists of patients with both a high baseline NT-proBNP (>1000 pg/L) and a persistently elevated sST2 ratio (>0.75), demonstrating how sST2 can reclassify risk when NT-proBNP alone is insufficient [79]. Rehman et al. also observed that in cohorts with low sST2 levels, natriuretic peptides lost their prognostic value for mortality, highlighting the interdependence of these pathways [78].

## 7. A Proposed Hypothesis-Generating Multimarker Framework for Heart Failure Management

**Figure 3:**
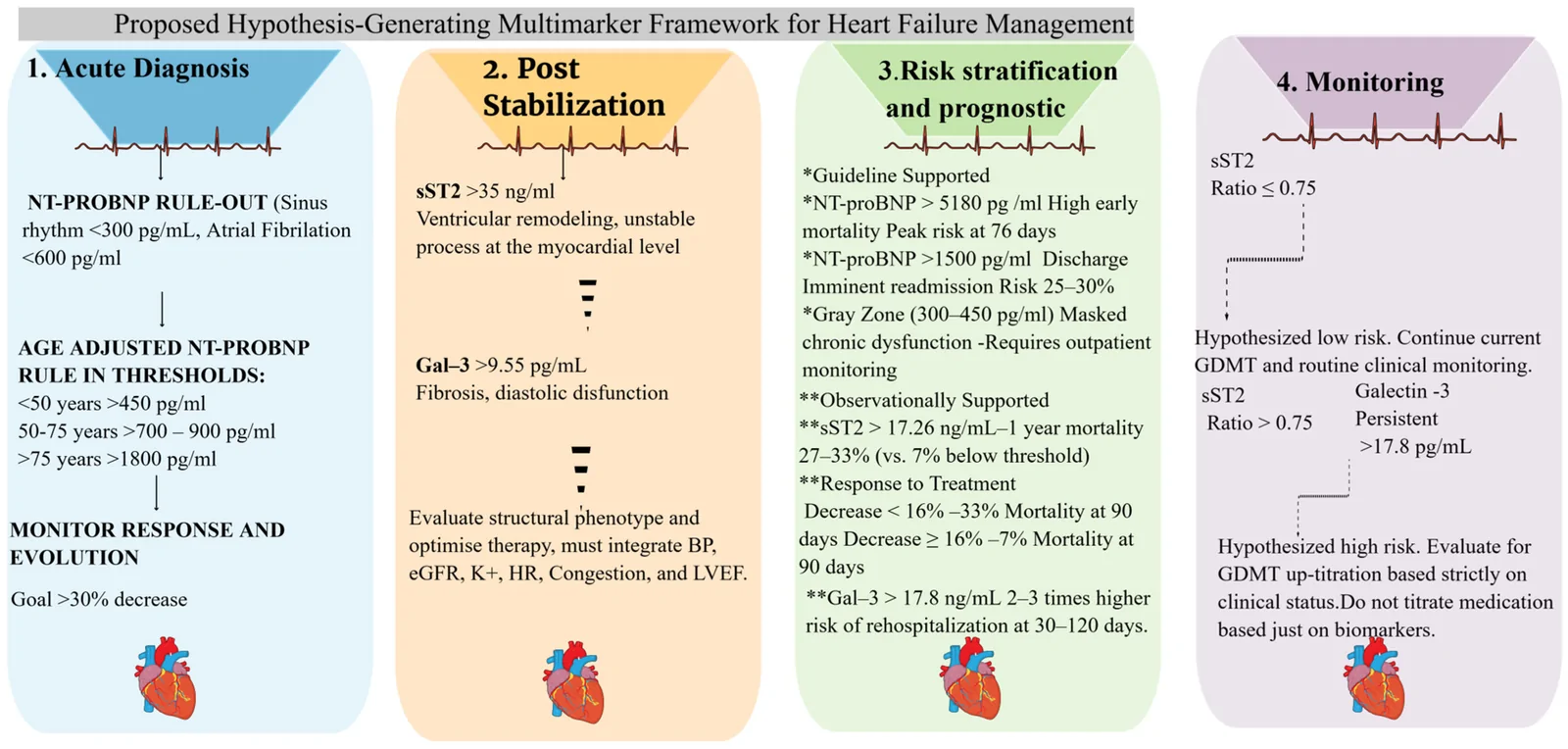
Proposed hypothesis-generating multimarker framework [29,78,86,87,88,89,90,91,92,93,94]. This diagram distinguishes guideline-supported elements from observationally supported and hypothetical elements. Clinical application caveat: This framework represents a set of testable research hypotheses and requires prospective validation in randomized clinical trials before routine clinical use. Direct medication-dose instructions cannot be based solely on sST2 or Gal values. All therapeutic decisions must stringently incorporate the patient’s status, tolerance to treatment, and concomitant comorbidities. Note on visual pathways: Solid lines represent established, guideline-supported diagnostic and therapeutic pathways (e.g., acute evaluation of NT-proBNP). The dotted lines denote hypothesis-generating observationally supported frameworks (e.g., sST2 and Gal-3 monitoring) that require strict clinical correlation and future prospective validation. Note: Biomarker concentrations are standardized throughout this manuscript in pg/mL (mathematically equivalent to ng/L) to maintain consistency. This conceptual framework is intended solely for prognostic risk stratification. It does not validate or recommend direct treatment intensification or active drug titration based on sST2 or Gal-3 values, as prospective outcome data supporting biomarker-guided therapy for these novel markers are currently absent. Abbreviations: BP, blood pressure; eGFR, estimated glomerular filtration rate; GDMT, guideline-directed medical therapy; HR, heart rate; LVEF, left ventricular ejection fraction. This figure is original.

Rather than a prescriptive clinical protocol, the integration of novel biomarkers with established natriuretic peptides is best viewed as a set of testable research hypotheses. This proposed hypothesis-generating multimarker framework (Figure 3) aims to distinguish guideline-supported practices from those based on observational data, highlighting areas that require prospective randomized validation before routine clinical implementation.

### 7.1. Diagnosis and Acute Exclusion (Guideline-Supported)

The initial assessment of acute dyspnea in the emergency department remains firmly grounded in guideline-supported natriuretic peptide tests. Standard commercial assays for BNP and NT-proBNP sampled at admission offer a sensitivity of more than 90% and a negative predictive value of >95% for acute heart failure.

For the rapid exclusion of acute heart failure, established thresholds of 100 pg/mL for BNP and 300 pg/mL for NT-proBNP are utilized. For diagnostic confirmation (rule-in), the ICON RELOADED study validated age-adjusted NT-proBNP thresholds: 450 pg/mL (<50 years), 900 pg/mL (50–75 years), and 1800 pg/mL (>75 years). These thresholds remain robust, though clinicians must integrate patient-specific comorbidities such as atrial fibrillation, renal dysfunction, or obesity (which requires lowering the diagnostic thresholds [24,87].

In the differential diagnosis of acute dyspnea, natriuretic peptides remain the clinical cornerstone, with Mueller et al. reporting an AUC of 0.92 for BNP [85]. Conversely, remodeling and fibrosis biomarkers (sST2 and Gal-3) demonstrate limited diagnostic utility in this acute setting (AUCs 0.57–0.63), reflecting their role in chronic structural assessment rather than acute hemodynamic variation. The dynamics of NT-proBNP during hospitalization (e.g., >30% reduction from admission to discharge) faithfully reflect a decrease in parietal stress in response to therapy. Persistent elevations indicate residual congestion and require therapeutic reassessment [29,95]. Observational data suggest sST2 and Gal-3 levels fluctuate less with diuretic-induced volume shifts, generating the hypothesis that they may reflect underlying structural stability rather than acute volume status [89,96].

### 7.2. Post-Stabilization (Observationally Supported Hypotheses)

Following hemodynamic stabilization, adjusting oral guideline-directed medical therapy requires rigorous clinical stratification. Observational findings suggest that Gal-3-based phenotyping helps isolate a high-risk, profibrotic profile of patients with HFpEF, offering enhanced prognostic stratification for adverse remodeling.

Cui et al. observed that a value >9.55 pg/mL at the post-stabilization stage identifies patients with more pronounced diastolic dysfunction, though this must be corroborated by echocardiography (e.g., the E/e′ ratio) [78]. Furthermore, an observational study by Kirk et al. suggested Gal-3 might identify a fibrotic phenotype responsive to mineralocorticoid receptor antagonists [97].

However, direct medication-dose instructions based just on Gal-3 or sST2 are not supported by current evidence. Any decision to initiate or up-titrate MRAs, ARNI, or beta-blockers must be completely driven by the patient’s blood pressure, heart rate, estimated glomerular filtration rate, serum potassium, congestion status, left ventricular ejection fraction, general tolerance to treatment, and concomitant comorbidities [90,98].

For sST2, observational data from the PROVE-HF and PROTECT cohorts hypothesize that elevated post-stabilization values (>35 pg/mL measured via the Presage ST2 assay in both acute discharge and chronic stable cohorts) signal ongoing myocardial vulnerability. Because sST2 appears less affected by obesity or the HFpEF phenotype, it has been hypothesized to serve as a complementary safety tool to identify high-risk patients who might have falsely reassuring levels of NT-proBNP [89,99,100].

### 7.3. Risk Stratification and Prognosis (Observationally Supported)

Risk stratification is heavily based on discharge and early outpatient biomarker sampling. The ICON study demonstrated that an elevated level of NT-proBNP (>5180 pg/mL) in mixed heart failure phenotypes is a strong independent predictor of short-term mortality (76 days), particularly when combined with eGFR < 60 mL/min/1.73 m^2^ [30,93]. For sST2, outpatient sampling identifies it as an independent risk factor. Patients with chronic heart failure who have values of sST2 in the upper percentiles (>17.26 pg/mL assessed via standard immunoassays in stable outpatient heart failure cohorts) exhibit lower cardiovascular survival rates. Maintaining discharge values above established standard cut-off points (e.g., 35 pg/mL measured via the Presage ST2 assay in both acute discharge and chronic stable cohorts) identifies patients with a significantly elevated 1-year mortality risk compared to those below the threshold. Furthermore, patients who do not show at least a 16% decrease (measured from admission to discharge in acute decompensated heart failure) in sST2 during acute treatment demonstrate a higher 90-day mortality risk [92,101]. Regarding Gal-3, data from the COACH and PRIDE cohorts suggest that discharge values above 17.8 pg/mL (evaluated at discharge in acute heart failure patients) identify mixed patients with a two- to three-fold higher likelihood of 30- to 120-day rehospitalization. Unlike natriuretic peptides, Gal-3 tends to remain stable over medium-term follow-up (up to 6 months), hypothesized to reflect established irreversible structural fibrosis [93,94].

### 7.4. Serial Monitoring (Hypothetical Framework)

The transition from acute to chronic management represents a critical window. Research by Bayés-Genís et al. suggests that dynamic serial monitoring, specifically an sST2 ratio (14-day outpatient follow-up value divided by the baseline admission value), provides superior prognostic precision over a single measurement [98]. A hypothesized threshold of 0.75 is often cited in the literature; a ratio ≤ 0.75 (a >25% reduction) correlates with biological response and lower short-term risk, whereas a ratio > 0.75 identifies persistent biomechanical stress [52,102].

It is critical to note that the STRONG-HF trial validated a high-intensity care strategy utilizing NT-proBNP and clinical congestion assessments to rapidly up-titrate guideline-directed medical therapy. It did not utilize or validate sST2 or Gal-3 for this purpose [37,99,103]. Therefore, the hypothesis that a high sST2 ratio or a post-discharge increase in Gal-3 (>15%) should trigger aggressive up-titration of specific drugs (like MRAs or ARNIs) remains strictly investigational [96]. In routine practice, any medication adjustments prompted by biomarker trends must unconditionally incorporate the clinical context.

Currently, the European Society of Cardiology and the American Heart Association guidelines do not assign a specific recommendation for the routine use of sST2 and Gal-3 in up-titrating heart failure therapies, unlike NT-proBNP, which has a Class I recommendation for diagnosis and prognosis.

## 8. Future Directions: Artificial Intelligence and Multi-Omics Integration

Looking forward, the clinical utility of sST2 and Gal-3 is poised to be enhanced by integration with emerging technologies. Machine learning algorithms could synthesize the serial kinetics of these biomarkers with advanced echocardiographic parameters (e.g., global longitudinal strain) to establish multidimensional hemodynamic-molecular risk scores [103].

Furthermore, the advancement of rapid point-of-care testing (POCT) platforms for sST2 could facilitate real-time risk stratification during ambulatory visits. Future clinical trials should investigate whether biomarker-guided optimization strategies, aiming to suppress biomarker concentrations toward optimal target thresholds, yield superior reverse remodeling compared to traditional symptom-directed management [104].

## 9. Negative, Neutral, and Contradictory Evidence

Despite the robust prognostic strength and pathophysiological rationale of a multimarker approach, several limitations, neutral findings, and contradictory pieces of evidence must be critically acknowledged [105].

The clinical benefit of biomarker-guided therapy remains uncertain. Landmark randomized controlled trials, such as GUIDE-IT, demonstrated that tailoring heart failure therapy specifically to lower NT-proBNP levels did not significantly improve overall clinical outcomes (such as time-to-first heart failure hospitalization or cardiovascular mortality) compared to standard optimal medical therapy [72,106].

For novel biomarkers like sST2 and Gal-3, prospective randomized outcome data proving that adjusting medication dosing based on specific biomarker cut-off points improves clinical endpoints is currently lacking. Elevated concentrations of Gal-3 or sST2 frequently identify patients with advanced, irreversible structural disease, such as established interstitial myocardial fibrosis, where standard pharmacological up-titration of medical therapy may attenuate clinical benefit [107]. Although these biomarkers can improve risk classification compared with traditional models, this benefit is often limited in clinical practice. A prognostic association does not equate to a proven therapeutic benefit, as detecting high risk via an elevated biomarker level does not necessarily imply that modifying treatment will change the patient’s course. Also, applying acute diagnostic thresholds to chronic monitoring environments introduces critical methodological errors and misinterpretation [49,74].

## 10. Strengths and Limitations

The present report should be considered a narrative review rather than a systematic review, which may affect reproducibility as it is inherently susceptible to selection bias. However, this is one of the first comprehensive works that proposes a hypothesis-generating multimarker algorithm specifically designed to optimize risk stratification and therapeutic titration both in the acute emergency setting and during long-term outpatient monitoring.

While previous studies have focused predominantly on the isolated prognostic value of single biomarkers at static time points, mostly evaluating natriuretic peptides at admission or discharge, our proposed algorithm introduces a multimarker framework. The clinical novelty of this integrated approach lies in its sequential evaluation across four critical clinical phases, bridging acute hemodynamic congestion with chronic structural remodeling. Furthermore, instead of using biomarkers merely as passive tools for risk stratification, this algorithm directly couples novel biomarker dynamics, providing a possible clinical pathway.

Nevertheless, several limitations must be acknowledged regarding the clinical implementation of this algorithm. First, formal quality assessment and risk-of-bias evaluation were not performed for the included studies, representing a primary methodological limitation of this narrative review. Moreover, the newly proposed multimarker pathway is derived from evidence obtained predominantly from retrospective studies, post hoc secondary analyses of clinical trials, and observational cohorts. Consequently, it has not yet been prospectively validated in an independent, large-scale randomized controlled trial.

Second, a major challenge stems from the substantial variability in diagnostic and prognostic cut-off values across the literature. This heterogeneity is driven by differences in laboratory assay platforms (e.g., variations between automated chemiluminescence immunoassays and manual ELISA kits for sST2 and Gal-3), which prevents universal standardization of the threshold values utilized in our framework.

Finally, population-specific confounding factors represent a significant constraint. The evidence supporting these biomarkers is derived from geographically and phenotypically diverse cohorts, ranging from Western populations in major clinical trial registries to specific Asian observational cohorts. Variations in baseline characteristics, such as the prevalence of chronic kidney disease, obesity, and the exact distribution of HFrEF versus HFpEF phenotypes, may alter the diagnostic accuracy and therapeutic response of the algorithm when applied to a broader, unselected global population.

While NT-proBNP-guided therapy has been evaluated in large trials, the clinical utility of actively titrating guideline-directed medical therapy based on sST2 or Gal-3 cut-offs remains unproven. No prospective randomized outcome trials have demonstrated that modifying medical management according to sST2 or Gal-3 changes improves hard clinical endpoints.

In conclusion, while these limitations highlight the critical need for standardizing biomarker thresholds, our proposed hypothesis-generating multimarker algorithm serves as a proactive clinical framework, bridging the gap between molecular research and personalized, high-intensity heart failure care.

## 11. Conclusions

The management of heart failure has reached a critical juncture in which clinical stability, as traditionally defined by physical examination and natriuretic peptide levels, no longer ensures long-term event-free survival. The synergistic application of sST2 and Gal-3 in relation to NT-proBNP addresses two other distinct, intertwined pathophysiological domains—myocardial mechanical strain and progressive interstitial fibrosis.

While the hypothesis-generating multimarker algorithm based on the synergistic use of sST2 and Gal-3 offers a promising framework for the personalized management of heart failure, it must be approached with caution and clinical prudence. Despite the robust pathophysiological rationale and supportive evidence from retrospective cohorts and post hoc analyses of major trials, several questions remain unanswered.

Furthermore, we must acknowledge that a ‘one-size-fits-all’ approach is rarely successful in the heterogeneous landscape of heart failure. The integration of sST2 and Gal-3 should not replace clinical judgment, but should rather serve as a complementary approach. Future research is mandatory to determine the cost-effectiveness of this high-intensity monitoring strategy and to define the optimal frequency of testing in the long-term outpatient setting.

Ultimately, we stand at the beginning of a more precise era in cardiology, but the path from ‘promising biomarker’ to ‘routine clinical standard’ is paved with continuous validation and rigorous scrutiny. Achieving biological stability remains our goal, yet the most effective way to reach it remains open to further scientific inquiry.

In conclusion, NT-proBNP, sST2, and Gal-3 offer complementary, robust prognostic insights reflecting distinct pathophysiological pathways in heart failure. Nevertheless, their current clinical utility is strictly confined to risk stratification and prognostic assessment. Because prospective randomized evidence proving outcome benefits from sST2- or Gal-3-guided treatment intensification is lacking, these markers should not be used as direct therapeutic intervention targets in routine clinical practice.

## Figures and Tables

**Table 1:** Cut-offs for NT-proBNP.

Category/Application	Cut-Off Value	Clinical Setting & Sampling Time	Phenotype	Assay Platform	Significance	Data Source
Non-Acute Rule-Out	<125 pg/mL	Outpatient Clinic/Stable Ambulatory	HFrEF & HFpEF	Automated chemiluminescence	Excludes heart failure	ESC guidelines [5]
Acute Rule-Out	<300 pg/mL	Emergency Dept./Immediate Admission	Acute decompensated heart failure	Standard immunoassays	Excludes acute heart failure	ICON study [24]
Acute Rule-In (<50 years)	>450 pg/mL	Emergency Dept./Immediate Admission	Acute heart failure (Age < 50 years)	Electrochemiluminescence immunoassay	Confirms acute HF diagnosis in younger patients	ICON study [24]
Acute Rule-In (50–75 years)	>900 pg/mL	Emergency Dept./Immediate Admission	Acute heart failure (Age 50–75 years)	Electrochemiluminescence immunoassay	Confirms acute HF diagnosis in intermediate age group	ICON study [24]
Acute Rule-In (>75 years)	>1800 pg/mL	Emergency Dept./Immediate Admission	Acute heart failure (Age > 75 years)	Electrochemiluminescence immunoassay	Confirms acute HF diagnosis accounting for age/renal decline	ICON study [24]
High Prognostic Risk	>5180 pg/mL	Acute Admission/Hospital Discharge	HFrEF & HFpEF	Standard automated immunoassays	Predicts 76-day short-term mortality (especially if eGFR < 60)	ICON study [24]

**Table 2:** Cut-offs for sST2 in heart failure. The Presage^®^ ST2 Assay was sourced from Critical Diagnostics, San Diego, CA, USA.

Category	Cut-Off (pg/mL)	Clinical Setting & Sampling Time	Phenotype	Assay Platform	Significance	Data Source
Standard	>35	Acute (Discharge) & Chronic (Stable OutPatient)	HFrEF & HFpEF	Presage^®^ ST2 Assay	Standard prognostic threshold identifying high risk for mortality/rehospitalization	Januzzi 2013 [53]
Women	>28	Chronic (Stable OutPatient)	HFrEF & HFpEF	Presage^®^ ST2 Assay/Standard ELISA	Increased long-term risk of death or heart failure hospitalization	BIOS Consortium [52]
Men	>31	Chronic (Stable OutPatient)	HFrEF & HFpEF	Presage^®^ ST2 Assay/Standard ELISA	Increased long-term risk of death or heart failure hospitalization	BIOS Consortium [52]

## Data Availability

No new data were created or analyzed in this study. Data sharing is not applicable to this article.

## Supplementary Materials

The following supporting information can be downloaded at https://www.mdpi.com/article/10.3390/ijms27167480/s1.

## Abbreviations

The following abbreviations are used in this manuscript:

HF	Heart Failure
NT-proBNP	N-terminal pro-B-type natriuretic peptide
sST2	Soluble suppression of tumorigenicity 2
Gal-3	Galectin-3
CAD	Coronary artery disease
WHO	World Health Organization
HFpEF	Heart failure with preserved ejection fraction
ANP	Atrial natriuretic peptide
RAAS	Renin–angiotensin–aldosterone system
ICD	Implantable cardioverter-defibrillator
HFrEF	Heart failure with reduced ejection fraction
HR	Hazard ratio
IL-33	Interleukin 33
NF-κB	Nuclear factor kappa-light-chain-enhancer of activated B cells
IκBα	Inhibitor of kappa B alpha
Ang II	Angiotensin II
NOX4	NADPH oxidase 4
CMR	Cardiac magnetic resonance
MRA	Mineralocorticoid receptor antagonist
