# Supplementary material for: The Clinical Value of NT-proBNP, sST2, and Galectin-3 in the Management of Heart Failure: From Diagnosis to Dynamic Monitoring: A Narrative Review
Source: Int J Mol Sci. 2026 Aug 21;27(16):7480. doi: 10.3390/ijms27167480 (PMC13513204; doi:10.3390/ijms27167480)
Supplement: Supplementary file 1 [file ijms-27-07480-s001.zip › ijms-4440790-supplementary.pdf]

Table S1. Comprehensive Evidence Summary of Studies

Study / Reference	Study Design	Sample Size (n)	Phenotype	Setting	Assay Platform	Threshold / Cut-off	Primary Endpoint	Effect Estimate	Adjustment Variables	Main Limitations
**ICON Study [24]**	Prospective Observational Cohort	1461	Mixed acute heart failure	Acute Emergency Department	Chemiluminescence Immunoassay (Elecsys)	Diagnostic: < 300 pg/mL (rule-out); Age-adjusted rule-in (450, 900, 1800 pg/mL). Prognostic: > 5,180 pg/mL combined with eGFR < 60 mL/min/1.73m[illegible]	Short-term mortality (76 days)	Strong independent predictor of short-term mortality	Age, renal function, baseline clinical risk covariates	Single acute time-point assessment; assay variation across centers.
**PARADIGM-HF [31]**	Post-hoc Clinical Trial Sub-study	Baseline (n = 2002), 1-month (n = 1936), 8-month (n = 1758)	Chronic HFrEF	Outpatient / Chronic	Standard Automated Immunoassays(sSt2)	None (linear relationships observed; no specific threshold associated with risk)	Cardiovascular death or heart failure hospitalization	Sacubitril/valsartan caused greater sST2 reductions vs. enalapril; 1-month sST2 changes independently associated with outcome risk	NT-proBNP, hs-TnT, and baseline predictors	Post-hoc analysis; restricted to symptomatic HFrEF patients.
**STRONG-HF[37]**	Prospective Randomized Clinical Trial	1078	Acute Heart Failure (Post-discharge)	Inpatient / Early Outpatient Follow-up	Routine Clinical Platforms (NT-proBNP)	Dynamic early changes at 1 week (decrease ≥ 30%, stable, or increase > 10%)	180-day all-cause death or heart failure readmission	HIC reduced 180-day events regardless of baseline NT-proBNP; early NT-proBNP increases guided diuretic adjustment and up-titration rate, resulting in	Clinical congestion scores, vital signs, background GDMT	Open-label design; focused specifically on rapid up-titration phase.

								equivalent 180-day outcomes (13.5% vs. 13.2%, $P = 0.93$)		
**RELAX-AHF[54]**	Randomized Clinical Trial Sub-study	1,161	Acute Heart Failure (with mild-to-moderate renal impairment)	Inpatient / Multicenter Clinical Trial	Multiplex / Standard Immunoassays (7 circulating biomarkers)	Serial tracking at baseline, days 2, 5, 14, and 60; day 2 optimal for NT-proBNP/cystatin-C, later points for sST2/hs-cTnT/GDF-15	180-day cardiovascular mortality prediction (C-index gain)	Multimarker combination (NT-proBNP, hs-cTnT, GDF-15, sST2) increased C-index by 0.08 to 0.87 (95% CI $0.83-0.91$)	Baseline biomarkers, clinical characteristics	Post-hoc sub-study analysis; trial inclusion criteria restrict generalizability (e.g., SBP > 125 mmHg).
**Cui et al.[81]**	Prospective Observational Cohort	247 (217 hospitalized HF patients and 30 controls)	HFpEF	Outpatient / Ambulatory	Immunoassay / Circulating Expression Detection Gal-3	ROC evaluation (Gal-3 AUC 0.819; NT-proBNP AUC 0.806; sST2 AUC 0.584)	1-year adverse events	Gal-3 superior to sST2 in distinguishing HFpEF; HR 2.33 (95% CI $1.72-2.94$) per 1 SD increase for adverse events)	Clinical factors and NT-proBNP	Single-center design; focused on hospitalized HFpEF cohort with 1-year follow-up.
**Rehman et al.[79]**	Prospective Registry Cohort	346	Acute Heart Failure (preserved & impaired LVEF)	Acute Presentation / Inpatient	Standard Immunoassay (sST2)	Elevated baseline levels (ROC AUC 0.71 for 1-year mortality)	1-year mortality	Independent predictor of mortality (HR 2.04, 95% CI $1.30-3.24$); synergistic prognostic value with natriuretic peptides	LVEF, age, NYHA class, renal function	Moderate sample size ($n = 346$); focused on acute setting.
**Mueller et al.[82]**	Prospective Head-to-Head	251 (137 acute heart failure, 114	Mixed heart failure	Emergency Department	Abbott Diagnostics (Gal-3, BNP) & Presage	ROC curve analysis	Acute diagnostic accuracy & 1-	BNP superior for acute diagnosis (AUC 0.92); Gal-3	Framingham score, echocard	Single emergency department setting;

	Cohort Study	non-cardiac dyspnea)			ST2 assay (sST2)		year all-cause mortality	(AUC 0.70) and sST2 (AUC 0.75) comparable to BNP (AUC 0.72) for 1-year mortality prediction	iographic findings	evaluation limited to dyspenic patients.

Note: This table highlights representative pivotal studies selected from the literature .

The following supporting information can be downloaded at https://www.mdpi.com/article/doi/s1.
